# Maternal Serum α-Fetoprotein Levels during Pregnancy and Testicular Cancer in Male Offspring: A Cohort Study within a Danish Pregnancy Screening Registry

**DOI:** 10.3390/ijerph192114112

**Published:** 2022-10-28

**Authors:** Cecilie S. Uldbjerg, Youn-Hee Lim, Clara H. Glazer, Russ Hauser, Anders Juul, Elvira V. Bräuner

**Affiliations:** 1 Department of Growth and Reproduction, Copenhagen University Hospital—Rigshospitalet, 2100 Copenhagen, Denmark; 2 The International Research and Research Training Centre in Endocrine Disruption of Male Reproduction and Child Health (EDMaRC), Rigshospitalet, University of Copenhagen, 2100 Copenhagen, Denmark; 3 Section of Environmental Health, Department of Public Health, University of Copenhagen, 2100 Copenhagen, Denmark; 4 Department of Preventive Medicine, Seoul National University College of Medicine, Seoul 03080, Korea; 5 Department of Urology, Zealand University Hospital, 4000 Roskilde, Denmark; 6 Department of Environmental Health, T.H. Chan School of Public Health, Harvard University, Cambridge, MA 02115, USA; 7 Department of Clinical Medicine, University of Copenhagen, 2100 Copenhagen, Denmark

**Keywords:** α-fetoprotein, serum, pregnancy, testicular cancer

## Abstract

Testicular cancer is believed to originate from disruptions of normal androgen-estrogen balance in-utero. α-fetoprotein (AFP) may modify fetal response to estrogens via estrogen interaction. In a cohort study, we investigated the association between circulating maternal pregnancy AFP and testicular cancer risk in offspring. Of the 56,709 live-born males from a pregnancy screening registry in 1980–1995, our study included 50,519 singleton males with available second trimester blood samples from their mothers and complete covariate ascertainment. Testicular cancer diagnoses and covariate data were obtained from nationwide Danish health registries. Cox regression and Kaplan–Meier analyses estimated the prospective risk of testicular cancer (all, seminoma, nonseminoma) by AFP multiples of the median. During follow-up, 163 (0.3%) of the included males developed testicular cancer, of which 89 (54.6%) were nonseminomas. Maternal serum AFP levels greater than/equal to the median were associated with a relative risk of testicular cancer close to unity (RR 1.04, 95% CI 0.76; 1.41) compared to AFP below the median. Associations differed by type of testicular cancer (RR_seminoma_ 0.81, 95% CI 0.51; 1.29, RR_nonseminoma_ 1.31, 95% CI 0.85; 2.02). On balance, our findings do not support that serum AFP in pregnancy can be used as a predictor of testicular cancer in offspring.

## 1. Introduction

Testicular cancer represents the most common malignant cancer in young males aged 15 to 44 years [1,2]. Testicular germ cell tumors can broadly be divided into two types, namely seminomas and nonseminomas [1]. Clinically, the two types have different histological and clinical characteristics, where nonseminomas tend to develop and spread more quickly, while seminomas often present later in life and spread more slowly [1,3,4]. It is widely acknowledged that both types have a fetal origin and are derived from germ cell neoplasia in situ (GCNIS) [1,5,6]. GCNIS is believed to reflect fetal gonocytes that failed to differentiate to spermatogonia and develop into the malignant GCNIS germ cells after puberty [1,2,5,7,8,9,10]. It has been hypothesized that disruptions of the normal androgen-estrogen balance during early fetal development, particularly in critical windows of fetal gonocyte development, may initiate the development of testicular cancer prenatally in the male fetus [9]. Otherwise, the specific etiology of testicular cancer is vaguely understood but established risk factors of testicular cancer include cryptorchidism, ethnicity, family testicular cancer history, being a twin and being small for gestational age among others [11,12].

α-Fetoprotein (AFP) is a glycoprotein that is mainly produced in the fetal yolk sac and fetal liver hepatocytes and excreted to amniotic fluid [13,14]. Malformations that decrease the integrity of the placental barrier, such as neural tube defects, omphalocele, gastroschisis and certain renal defects, are recognized to increase the transfer of AFP (and other fetal substances) from amniotic fluid to maternal serum, resulting in a subsequent increase in maternal serum AFP levels [13]. For this reason, serum AFP has been used as a diagnostic marker for fetal developmental diseases in Denmark for over 40 years [13]. Although it is recognized that AFP plays an important role in fetal development, the exact mechanisms are not fully elucidated [13,15,16]. However, it has been postulated that AFP can modify the fetal response to estrogens via estrogen interaction. The specific AFP-estrogen interaction and the mechanism by which AFP impair the fetal response to estrogens likely involve processes, where estradiol induces conformational changes in AFP through exposition of a growth inhibitory protein segment that is recognized as an estrogen binding site [17,18,19,20]. This process could change the availability of estrogen and in turn potentially affect the intrauterine androgen-estrogen balance and the subsequent risk of testicular cancer in the offspring. Alternatively, the disruption of the normal placental function and a reduced integrity of the placental barrier due to fetal malformations could lead to a higher mother-to-fetus transport of pregnancy hormones and other endocrine disruptors [17,18,19], why AFP levels in this context may represent an indirect marker of the possible contribution of other factors to the risk of testicular cancer in later life.

In our study, we aimed to investigate the prospective association between circulating maternal AFP levels in second trimester serum and the risk of testicular cancer in male singleton offspring.

## 2. Materials and Methods

### 2.1. Study Design and Data Source

We used a cohort study design of singleton males born to Danish mothers from a Danish Pregnancy Screening Registry, a large cohort of pregnant women who participated in a screening program in the period 1980–1995. As part of the screening, maternal serum AFP was measured and recorded during second trimester of pregnancy (gestational weeks 14 to 22) and data are now maintained at the Danish National Biobank (State Serum Institute in Copenhagen, Denmark). Outcome (testicular cancer) and covariate data were obtained from the nationwide Danish health registries with complete coverage. In Denmark, all residents have been provided a unique CPR number since the establishment of the Danish Civil Registration System (CRS) in 1968, which allows for accurate linkage between registries [21,22].

### 2.2. Study Population

**Figure 1:**
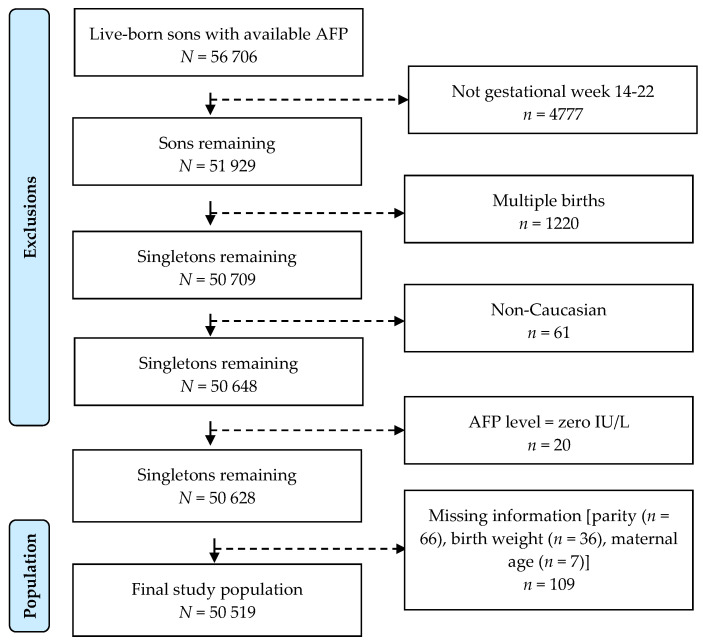
Flowchart of the study population.

The sample population consisted of 56,706 live-born sons born to mothers from a Danish Pregnancy Screening Registry. We excluded sons of mothers who did not provide serum samples within the optimal period for measuring AFP levels in gestational weeks 14–22 (*n* = 4777), sons who were twins (*n* = 1220) and sons who were non-Caucasian (*n* = 61). Of these, we further excluded those with AFP values of zero (*n* = 20) and missing covariate information (*n* = 109: parity (*n* = 66), birth weight (*n* = 36), maternal age (*n* = 7)). The final study population included 50,519 live-born singleton sons with complete covariate ascertainment (Figure 1).

### 2.3. Testicular Cancer

Incident testicular cancer were identified using the nationwide Danish Cancer Registry (DCR), which is a population-based registry containing data on the incidences of all cancers since 1943 [23]. Testicular cancer has unmistakable features, why cases are unlikely to be missed or go undiagnosed. The DCR has adopted the International Classification of Diseases for Oncology, third edition (ICD-O-3) [24] coding in 2004, and registrations from 1978–2003 have also been converted to ICD-O-3. All testicular cancers are described according to topography (C62) and morphology codes (classical seminoma: 9060–9062, 9063, 9064; nonseminoma without mixed germ cell tumors (MGCT): (9065, 9070–9072, 9080–9084, 9100–9102) and MGCT: (9085). In the present study, we applied two strata, namely classical seminoma and nonseminoma (including MGCT). Sons were followed for information on vital status and emigration in the CRS until 31 December 2020. Two males that were diagnosed with rare embryonal rhabdomyosarcoma of the genitals (topography C62 and morphology code 89103) at age < 4 years were censored on the date of that diagnosis.

### 2.4. α-Fetoprotein (AFP)

In Denmark, AFP measurements in maternal serum have previously been offered to all pregnant women since 1978, and a screening program was established in 1980. Information recorded at the time of screening included the woman’s unique CPR number, date of birth, maternal serum AFP level, test date and gestational age at the time of testing. We standardized all AFP levels in maternal serum to gestational age because it is recognized that AFP in maternal serum is largely dependent on gestational age [25]; as also observed in our study population. This was done by dividing the absolute AFP maternal serum level by the median AFP serum level in all measurements from singleton births taken at the same gestational age in the same calendar year (which gives multiples of the median [MoM]) [25].

### 2.5. Covariates

Data on relevant covariates were obtained from the Medical Birth Registry (maternal and birth outcomes), the DCR (parental history of testicular cancer (C62)) and the National Patient Registry (cryptorchidism (ICD8: 75210, 75211, 75219/ICD10: Q53, Q531[A], Q532[A], Q539)). Covariates were identified *a priori* based on existing knowledge in the literature: Maternal age (<25, 25–29, 30–34, 35–39, ≥40 years), birth weight for gestational age [small for gestational age (SGA, <10th percentile), normal (10th–90th percentile), large for gestational age (LGA, >90th percentile for males of the same GA], parity (0, 1, ≥2), cryptorchidism in sons (no, yes), paternal history of testicular cancer (no, yes).

### 2.6. Statistical Analyses

We calculated descriptive statistics to identify frequencies of characteristics in the study population stratified according to testicular cancer diagnosis (overall, seminoma, nonseminoma (incl. MGCT)). We used Cox proportional hazards regression models with attained age as the underlying time scale to investigate testicular cancer (overall, seminoma, nonseminoma (incl. MGCT)) as a function of serum AFP (represented by MoM categories), expressed as relative risks (RR). All males contributed person-time (years under observation) from the date of birth until an incident diagnosis of testicular cancer, death, emigration or the end of follow-up on 31 December 2020, whichever came first. The associations between AFP and risk of testicular cancer were estimated with increasing levels of adjustment: Model 1 was adjusted for son’s age (underlying timescale) and baseline birth year; Model 2 (used as the main model) was further adjusted for maternal and birth outcomes (including birth weight for gestational age, parity and maternal age) and paternal history of testicular cancer; and Model 3 was further adjusted for cryptorchidism, as the disorder is directly related to the testicular dysgenesis and we sought to investigate how this variable potentially affected our main model [11]. AFP levels were categorized below and above/equal to the median (<1.00 (the referent group); ≥1.00 MoM).

Kaplan–Meier plots were used to illustrate time course free of testicular cancer for all, seminoma and nonseminoma (incl. MGCT) testicular cancer, using age (years) as the horizontal (x) axis and probability of not being diagnosed with testicular cancer (proportion of males without testicular cancer) as the vertical (y) axis, according to overall MoM and stratified according to MoM below and above/equal to the median (<1.00/≥1.00).

To examine effect modification and the trend of the RR of testicular cancer according to AFP levels (<1.00; ≥1.00 MoM) across the levels of each of the included covariates, we included an interaction term between serum AFP and the covariate. We used the likelihood ratio test for each interaction in Model 2, but with no adjustment for the specific included covariate (interaction variable).

All statistical analyses and graphical presentations were produced in R version 4.0.3 (R Core Team, Vienna, Austria, 2016), using the following packages: dplyr, stats, survival, splines, mets, gplots, plotCI and Hmisc. All statistical tests were two-sided, and *p*-values < 0.05 were considered statistically significant.

The manuscript follows the Strengthening the Reporting of Observational Studies in Epidemiology (STROBE) statement and relevant checklist [26].

### 2.7. Ethical Considerations and Data Protection

The authors assert that all procedures contributing to this research comply with the ethical standards of the relevant national laws and according to the Helsinki Declaration. The present study was approved by the Danish Data Protection Agency (J.nr. RH-2018-59, suite nr. 06306, with updates in Pactius P-202-1007) and the local Danish Ethical committee (J.nr. 17030851). According to Danish law, data from national screening programs can be used for research without obtaining individual informed consent.

## 3. Results

The distribution of descriptive characteristics of the cohort of males are presented in Table 1. The total follow-up time was 1,613,375 person-years. During follow-up, 163 (0.3%) of the 50,519 included males developed testicular cancer (seminoma/nonseminoma (incl. MGCT)/not classified due to missing morphology code: 73/89/1). The mixed germ cell tumors, MGCT, represented 22 cases (24.7%) of identified nonseminomas (not shown in tables). Compared to males without testicular cancer, males with testicular cancer were generally more likely to be premature, be small for gestational age, be born to mothers below age 25 years and have cryptorchidism, whilst parity was similar amongst the two groups. Among the sub-groups of testicular cancer, males were diagnosed with seminoma at an older age (median: 28.7 years) than males with nonseminoma (median: 24.9 years). Males with seminoma were more likely to be small for gestational age, have cryptorchidism, be born to term and be born earlier in the study period (year 1980–1984) than males with nonseminoma.

The associations between maternal serum AFP in second trimester and risk of testicular cancer in male offspring are provided in Table 2. Overall, none of the observed associations were statistically significant. In main adjusted analyses, males in the MoM categories of maternal AFP serum levels above or equal to the median (≥1 MoM) had a relative risk of testicular cancer close to unity (RR 1.04, 95% CI 0.76; 1.41) compared to males in the reference category under the median (<1 MoM). Considering types of testicular cancer, a higher risk was observed for nonseminomas (RR 1.31, 95% CI 0.85; 2.02), but not for classical seminomas (RR 0.81, 95% CI 0.51; 1.29). The respective point estimates for seminomas and nonseminomas were outside the confidence intervals for the other. Additional adjustment of cryptorchidism in the analyses did not change the estimates. In further investigations of the dose–response effect between MoM quartile categories of maternal AFP serum levels and testicular cancer risk, we detected a U-shaped response for seminomas, but an increasing response over the first three quartiles to a plateau for nonseminomas, albeit with non-significant trend estimates (Table S1).

**Figure 2:**
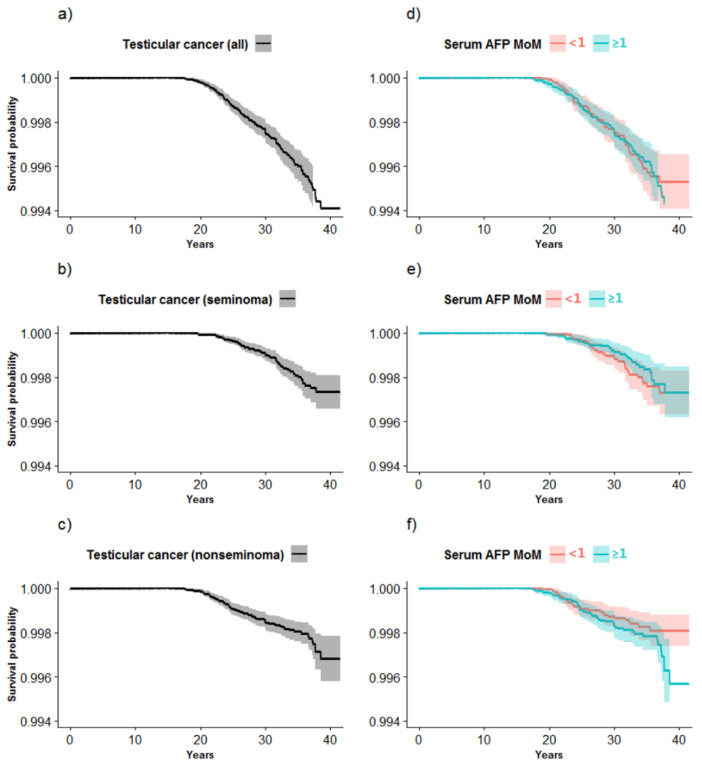
(**a**–**f**). Kaplan–Meier plots for time course free of all, seminoma and nonseminoma (incl. mixed germ cell tumors) testicular cancer (**a**–**c**) and plots stratified according to serum α-fetoprotein (AFP) multiples of the median (MoM: <1.00/≥1.00) for (**d**) all, (**e**) seminoma and (**f**) nonseminoma testicular cancer.

The time course with no testicular cancer (all, seminoma, nonseminoma (incl. MGCT)) is illustrated in Kaplan–Meier plots overall (Figure 2a–c) and according to AFP maternal exposures above and below the median for (d) all, (e) seminoma and (f) nonseminoma. The latest diagnosis of all testicular cancers was made at age 39 years (mean: 27.4 years) and nonseminomas were diagnosed at an earlier age (mean: 26.2 years) than seminomas (mean: 28.7 years), (Figure 2a–c). It is visually evident that the male offspring exposed to AFP above/equal to and below the median AFP follow similar time courses for all testicular cancers. In comparison, differing time courses are observed for seminomas (longer time course) and nonseminomas (shorter time course) according to AFP above/equal to the median compared to below the median (Figure 2d–f).

The results of the effect modification analysis are presented in Table 3. None of the included covariates modified the association between maternal AFP levels and testicular cancer; overall (P_interaction_ ≥ 0.137), seminoma (P_interaction_ ≥ 0.124) or nonseminoma (P_interaction_ ≥ 0.060).

## 4. Discussion

In this cohort study of 50,592 males, maternal serum AFP levels during pregnancy were not associated with the overall risk of testicular cancer in male offspring. Noteworthy, an increased risk of nonseminomas according to increased AFP was observed, but the association was subject to large statistical uncertainties.

To our knowledge, only one previous study by Zhang et al. has attempted to examine this association by comparing maternal AFP in different ethnic female populations with known high risk (white populations) and low risk (black populations) of testicular germ cell cancer based on maternal blood samples drawn in the early 1960s [27]. With this methodological approach, the authors used race as a proxy of testicular cancer and solely relied on the assumption that whites were at higher risk of testicular cancer but failed to directly ascertain the testicular cancer status of the male offspring. They detected higher AFP levels in the low-risk group of women and concluded that higher maternal AFP levels are thus associated with a reduced risk of testicular cancer. However, such comparisons based entirely on ethnicity are rather complex as the lower incidence of testicular cancer among black populations may partly be explained by lower access to health care and limited screening options in minority populations rather than a biological explanation [28], which seems plausible as lower survival rates of testicular cancer have been observed among black males [29]. Furthermore, as blood samples were drawn during a time of great racial inequality in the US, the applicability to a contemporary cohort is uncertain due to potential recruitment bias during that period, where black populations had less access to health services.

In 2001, we proposed that testicular cancer was part of a syndrome named *testicular dysgenesis syndrome* (TDS) that also comprises cryptorchidism, hypospadias and poor semen quality [30]. It is thus noteworthy that a previous study based on a smaller group of males from the same cohort (*n* = 25,418) reported a 63% greater risk of cryptorchidism in male offspring associated with high maternal AFP during pregnancy (≥2.5 times the multiple of median) compared to low maternal AFP (within 25% of the median) [31]. However, those estimates were also statistically non-significant, and it remains unclear from both our present study and that previous study whether the non-significant results are due to a lack of an association or rather a lack of statistical power.

Serum AFP is a well-established diagnostic biomarker in a clinical context, both to reveal testicular cancer in male serum and to detect developmental anomalies in the fetus during pregnancy in maternal serum. For instance, in nonseminoma testicular cancers, the tumors can produce abnormally high levels of AFP (mainly from embryonal carcinoma and yolk sac carcinoma). These levels of AFP in nonseminoma testicular cancer patients have been proven directly proportional with tumor growths, why AFP levels provide important prognostic information and have been incorporated into staging criteria used to group patients into risk groups (good, intermediate, poor) according to the International Germ Cell Consensus Classification [32,33]. Although we were curious to whether a similar link between maternal AFP levels during pregnancy and testicular cancer in offspring was evident, our study does not provide strong evidence supporting such an association. Intriguingly, we observed differential effects of maternal AFP during pregnancy when considering types of testicular cancer, with respective positive and negative associations for nonseminoma and seminomas. It is important to note that the point estimates for nonseminoma and seminoma were not within the confidence intervals for the other, suggesting markedly different associations with AFP. Particularly, the decreased risk for seminomas was rather surprising but likely a result of an incomplete case ascertainment due to a relatively short observation time, for which most seminoma cancers are not identified yet. An increased risk for nonseminomas may be evident, but at the same time, the association was subject to considerable statistical uncertainty and therefore largely speculative. This indication of a possible modifying effect of testicular cancer type needs to be clarified on in other cohorts.

### Strengths and Limitations

A key strength of our study is the relatively large cohort and use of nationwide health registries known for validity and completeness in reporting. We used a prospective cohort design with limited potential for any bias (differential misclassification/selection), as all our covariate and outcome registry data were acquired independently of AFP maternal levels and thus not dependent on recall. Health care is free in Denmark so socio-economic status would not influence whether or not persons seek medical health and ultimately appear in health registry records. We were able to include important confounders with access to information on cryptorchidism and paternal history of testicular cancer, which are both established risk factors for testicular cancer. We also had access to a unique collection of maternal serum AFP measurements obtained during the critical time window of the etiologic relevance for testicular cancer. To account for variation of AFP levels during pregnancy, we standardized AFP levels by gestational age. We restricted our cohort to singleton sons because mothers of twins (or other multiple births) typically have at least twice the level of serum AFP during pregnancy than a mother carrying only one child, and the same may cause a differential risk of testicular cancer in offspring [34]. Therefore, the potential confounding effect of multiple births was avoided in our analyses.

Finally, the vast majority of the Danish population is Caucasian, and we excluded Greenlandic women who may have a different risk of testicular cancer in offspring, why we overall do not expect any confounding by ethnicity in our study.

However, the study also has some limitations. A limitation is the lack of repeated AFP quantification in each serum sample that would ensure better precision of AFP levels of each woman. Although we included a large population of over 50,000 pregnant women, the observation time was insufficient since the male offspring only had a maximum follow-up time of 40 years (total person years: 1,613,375). This means that we were most likely unable to identify all cases of testicular cancer, and particularly seminomas, as this type is usually diagnosed in older men and may occur after end of follow up. Of the 163 cases of testicular cancer identified in our cohort, the proportion of seminomas (45%) was relatively low compared to the general population (accounting for approximately 55% [35]) and the median age of males with seminoma (28.7 years) was younger than that found in cohorts followed up to an old age [36,37,38]. This implies that a longer follow-up period may in fact have led to the inclusion of more case males diagnosed with testicular cancer (mainly seminomas) and possibly providing greater statistical power and better precision in our estimates of associations. Incident testicular cancers were identified using the nationwide DCR, known for completeness and validity, but we acknowledge that inclusion of MGCT in the class of nonseminomas may be associated with some misclassification, as the MGCT morphology code possibly includes *all* mixed histologies, regardless of whether tumors are with combinations of nonseminoma histologies or tumors with both seminomatous and nonseminomatous elements. We censored cases at the time of their incident testicular cancer diagnosis, thus in the case of incident unilateral testicular cancer, we are unable to assess whether or not potential later diagnoses in the other testicle were of the same morphological group.

Although we restricted our analyses to singleton pregnancies, we were not able to make further exclusions based on relevant determinants of AFP. High AFP levels may also occur in other conditions besides twin gestation such as fetal abdominal wall defects, placental anomalies and pregnant women with autoimmune diseases among others, but unfortunately, we did not have access to this information. Nevertheless, we do not expect this to cause significant concerns to the overall interpretation of findings, as these mentioned conditions are relatively rare and likely to not confound or mediate the association between AFP and testicular cancer, as they are not identified as risk factors of testicular cancer.

The pregnant women in our study cohort may not necessarily be representative of the general pregnant population in Denmark. An important concern with study populations recruited from pregnancy screening programs is that these are already identified as high-risk pregnancies given the indication for pregnancy screen. As the main indication was high maternal age, the women in our study population were slightly older than otherwise expected at the time of data collection, but we attempted to account for this in our models by adjusting for maternal age. Unfortunately, we did not have access to data on maternal morbidities at the time of sample collection to assess if underlying maternal diseases could contribute to any observed associations. In addition, another source of bias may be that the women who are generally willing to consent to data being used in research cohorts are known to be more educated or from a higher social class than those not consenting. Despite these proposed small differences as well as adjustment for maternal age in our analyses, our findings may have limited generalizability. Finally, since AFP is used for screening of malformations and chromosomal malformations, women with abnormally high AFP levels may have chosen to induce abortion, why sons of these women with very high AFP values are likely not included in the study population, leading to some missed cases.

## 5. Conclusions

Using a large prospective cohort design, maternal serum α-fetoprotein levels during pregnancy were not associated with the overall risk of testicular cancer in the offspring. An increased risk for nonseminomas and a possible modifying effect of cancer type is largely speculative and remains inconclusive. Therefore, our findings do not provide evidence supporting that serum α-fetoprotein in pregnancy can be used as a predictor of testicular cancer in later life. Further studies need to clarify on the association.

## Figures and Tables

**Table 1:** Distribution of factors characterizing the cohort of 50,519 Danish singleton males and their mothers who participated in a maternal second trimester serum α-fetoprotein (AFP) screening program between 1980 and 1995.

Variable	Non-Cases, *n* (%)	Testicular Cancer Cases, *n* (%)		
	(*n* = 50,354)	All (*n* = 163) ^#^	Seminoma (*n* = 73) ‡	Nonseminoma (*n* = 89) ‡^,^*
Son’s age at testicular cancer diagnosis, median (25th–75th percentile)				
	-	26.5 (23.6–31.6)	28.7 (25.4–32.1)	24.9 (22.4–29.9)
Gestational age (weeks) at birth				
≤39	18,994 (37.7)	69 (42.3)	24 (32.9)	44 (49.4)
40	17,855 (35.5)	55 (33.7)	25 (34.2)	30 (33.7)
≥41	13,505 (26.8)	39 (23.9)	24 (32.9)	15 (16.9)
Preterm (<37 weeks)				
Yes	2473 (4.9)	9 (5.5)	3 (4.0)	6 (6.4)
Birth weight (g)				
≤2999	7519 (14.9)	24 (14.7)	10 (13.7)	13 (14.6)
3000–3499	15,832 (31.4)	52 (31.9)	24 (32.9)	28 (31.5)
3500–3999	17,421 (34.6)	56 (34.4)	26 (35.6)	30 (33.7)
4000–4499	7825 (15.5)	27 (16.6)	11 (15.1)	16 (18.0)
≥4500	1757 (3.5)	4 (2.5)	2 (2.7)	2 (2.3)
Birth weight for gestational age				
SGA ^a^	5005 (9.9)	19 (11.7)	9 (12.3)	9 (10.1)
Normal ^b^	39,094 (77.6)	125 (76.7)	59 (80.8)	66 (74.2)
LGA ^c^	6285 (12.5)	19 (11.7)	5 (6.9)	14 (15.7)
Birth year of son				
1980–1984	11,205 (22.3)	51 (31.3)	26 (35.6)	24 (27.0)
1985–1989	21,082 (41.9)	82 (50.3)	34 (46.6)	48 (53.9)
1990–1996	18,067 (35.9)	30 (18.4)	13 (17.8)	17 (19.1)
Maternal parity (no. of births prior to index birth)				
0	24,373 (48.4)	78 (47.9)	33 (45.2)	44 (49.4)
1	17,179 (34.1)	56 (34.4)	28 (38.4)	28 (31.5)
≥2	8802 (17.5)	29 (17.8)	12 (16.4)	17 (19.1)
Maternal age (years) at birth				
<25	11,637 (23.1)	41 (25.2)	15 (20.5)	26 (29.2)
25–29	19,003 (37.7)	56 (34.4)	29 (39.7)	26 (29.2)
30–34	11,881 (23.6)	41 (25.2)	18 (24.7)	23 (25.8)
35–39	6801 (13.5)	20 (12.3)	10 (13.7)	10 (11.2)
≥40	1032 (2.1)	5 (3.1)	1 (1.4)	4 (4.5)
Cryptorchidism in sons				
Yes	1874 (3.7)	12 (7.4)	6 (8.2)	6 (6.7)
Paternal testicular cancer				
Yes	52 (0.1)	1 (0.6)	1 (1.4)	0 (0.0)

^a^ Small for gestational age (GA) (<10th percentile for males of the same GA); ^b^ Normal for GA (10th–90th percentile for males of the same GA); ^c^ Large for GA (>90th percentile for males of the same GA); ^#^ Of the 165 identified testicular cancer (C62) cases, two were censored as their diagnosis was given at ages < 4 years and would not be true testicular cancer; ‡ Note: ICD-0–3 morphology codes were missing for one testicular cancer case, why these are not classified into seminoma/nonseminoma; * Nonseminomas included 22 cases with mixed germ cell tumors.

**Table 2:** Relative risk (RR) of testicular cancer in male offspring according to serum levels of α-fetoprotein (AFP) during the second trimester of pregnancy.

Testicular Cancer	AFP MoM ^a^	Ncases/Person Years ^b^	Relative Risk (95% CI)		
			Model 1 ^c^	Model 2 (Main) ^d^	Model 3 ^e^
All	<1.00	73/749957	1 (reference)	1 (reference)	1 (reference)
	≥1.00	90/863418	1.06 (0.78; 1.44)	1.04 (0.76; 1.41)	1.03 (0.76; 1.41)
Seminoma	<1.00	38/749063	1 (reference)	1 (reference)	1 (reference)
	≥1.00	35/861942	0.79 (0.50; 1.24)	0.81 (0.51; 1.29)	0.81 (0.51; 1.29)
Nonseminoma *	<1.00	34/748839	1 (reference)	1 (reference)	1 (reference)
	≥1.00	55/862408	1.40 (0.91; 2.15)	1.31 (0.85; 2.02)	1.31 (0.85; 2.01)

^a^ AFP levels in maternal serum were standardized to gestational age by dividing the absolute value by the median value across all singleton live births, for each gestational week and for each calendar year to give multiples of the median (MoM); ^b^ Years of observation for the entire cohort during follow-up; ^c^ Model 1: Adjusted for age as the underlying timeline and baseline year; ^d^ Model 2: Model 1 + birth weight for gestational age, parity, maternal age, paternal testicular cancer; ^e^ Model 3: Model 2 + cryptorchidism; * Nonseminomas included mixed germ cell tumours (*n* = 22).

**Table 3:** Serum AFP ** and risk of testicular cancer (all) in offspring stratified by age, reproductive variables and other covariates in the cohort of 50,519 Danish singleton males and mothers stratified by AFP multiples of the median (MoM).

	Overall		Seminoma		Nonseminoma *	
Stratification Variable	RR for AFP MoM ≥ 1 vs. AFP MoM < 1 (95% CI) ‡	P for Trend	RR for AFP MoM ≥ 1 vs. AFP MoM < 1 (95% CI) ‡	P for Trend	RR for AFP MoM ≥ 1 vs. AFP MoM < 1 (95% CI) ‡	P for Trend
Gestational age (weeks) at birth						
≤39	1.16 (0.71; 1.90)	0.420	1.09 (0.48; 2.50)	0.120	1.28 (0.69; 2.40)	0.992
40	0.78 (0.46; 1.32)		0.41 (0.18; 0.94)		1.31 (0.63; 2.72)	
≥41	1.27 (0.68; 2.39)		1.21 (0.54; 2.70)		1.38 (0.50; 3.82)	
Preterm (<37 weeks)						
Yes	0.42 (0.11; 1.55)	0.377	0.26 (0.02; 2.85)	0.612	0.52 (0.11; 2.59)	0.513
No	1.09 (0.79; 1.50)		0.85 (0.53; 1.37)		1.40 (0.89; 2.19)	
Birth weight (g)						
<2500	0.48 (0.10; 2.36)	0.773	0.48 (0.03; 7.68)	0.924	na	0.557
2500–2999	0.63 (0.25; 1.60)		0.39 (0.09; 1.64)		0.94 (0.26; 3.33)	
3000–3499	1.23 (0.70; 2.15)		0.94 (0.42; 2.11)		1.58 (0.71; 3.49)	
3500–3999	0.86 (0.51; 1.46)		0.83 (0.38; 1.80)		0.89 (0.43; 1.83)	
4000–4499	1.84 (0.84; 4.02)		1.35 (0.41; 4.44)		2.29 (0.80; 6.61)	
≥4500	1.29 (0.18; 9.13)		na		na	
Birth weight for gestational age						
SGA ^a^	0.47 (0.19; 1.17)	0.478	0.54 (0.14; 2.00)	0.350	0.50 (0.14; 1.88)	0.318
Normal ^b^	1.14 (0.80; 1.63)		0.92 (0.55; 1.54)		1.41 (0.85; 2.33)	
LGA ^c^	1.20 (0.49; 2.94)		0.28 (0.03; 2.51)		1.86 (0.62; 5.57)	
Birth year of son						
1980–1984	1.59 (0.89; 2.82)	0.129	1.25 (0.57; 2.72)	0.692	2.49 (0.99; 6.29)	0.060
1985–1989	0.87 (0.56; 1.34)		0.58 (0.29; 1.15)		1.16 (0.65; 2.08)	
1990–1996	0.79 (0.39; 1.62)		0.81 (0.27; 2.43)		0.77 (0.30; 2.01)	
Maternal parity (no. of births prior to index birth)						
0	0.85 (0.54; 1.33)	0.491	0.67 (0.34; 1.33)	0.740	1.07 (0.59; 1.96)	0.600
1	1.25 (0.73; 2.13)		0.91 (0.43; 1.92)		1.78 (0.80; 3.93)	
≥2	1.22 (0.59; 2.55)		1.06 (0.34; 3.28)		1.36 (0.52; 3.59)	
Maternal age (years) at birth						
<25	1.07 (0.58; 2.00)	0.730	0.91 (0.33; 2.52)	0.759	1.18 (0.53; 2.60)	0.447
25–29	0.83 (0.49; 1.40)		0.70 (0.34; 1.46)		1.09 (0.50; 2.39)	
30–34	1.23 (0.66; 2.30)		0.73 (0.29; 1.86)		1.93 (0.79; 4.70)	
35–39	1.47 (0.60; 3.60)		1.03 (0.30; 3.56)		2.21 (0.57; 8.56)	
≥40	0.58 (0.10; 3.48)		na		0.29 (0.03; 2.75)	
Cryptorchidism in sons						
Yes	1.00 (0.32; 3.15)	0.151	0.74 (0.15; 3.67)	0.251	1.39 (0.26; 7.62)	0.514
No	1.03 (0.75; 1.43)		0.81 (0.50; 1.32)		1.30 (0.83; 2.03)	
Paternal testicular cancer						
Yes	na	na	na	na	na	na
No	1.02 (0.75; 1.40)		0.79 (0.49; 1.26)		1.31 (0.85; 2.02)	

Note: Testicular cancer rates in males were compared within the levels of each strata and males with the strata < 1 MoM served as the reference group; ** Maternal serum AFP levels were standardized to gestational age by dividing the absolute value by the median value across all singleton live births, for each gestational week and for each calendar year to give multiples of the median (MoM); * Nonseminomas included mixed germ cell tumors (*n* = 22); ‡ Adjusted for age (underlying timeline), baseline year, parity, maternal age at birth, paternal TC (unless the variable was stratified); ^a^ Small for gestational age (GA) (<10th percentile for males of the same GA); ^b^ Normal for GA (10th to 90th percentile for males of the same GA); ^c^ Large for GA (>90th percentile for males of the same GA).

## Data Availability

Data used in the present study are governed and maintained centrally by the Danish National Biobank, Statens Serum Institut and the Danish Data Health Authority. Data access is regulated by the EU General Data Protection Regulations (GPDR). Data are not publicly available and pseudo-anonymized data can only be accessed after approval by the Danish Data Health Authority and the Danish Data Protection Agency. Further details and other data that support the findings of this study are available from the corresponding author upon request.

## Supplementary Materials

The following supporting information can be downloaded at: https://www.mdpi.com/article/10.3390/ijerph192114112/s1, Table S1: Sensitivity analysis of trend, relative risk (RR) of testicular cancer (all) in male offspring according to serum levels of α-fetoprotein (AFP) during the second trimester of pregnancy.
